# The Effects of the Flash Technique Compared to Those of an Abbreviated Eye Movement Desensitization and Reprocessing Therapy Protocol on the Emotionality and Vividness of Aversive Memories

**DOI:** 10.3389/fpsyg.2021.741163

**Published:** 2021-12-23

**Authors:** Thomas C. Brouwers, Ad de Jongh, Suzy J. M. A. Matthijssen

**Affiliations:** ^1^Altrecht Academic Anxiety Centre, Altrecht GGz, Utrecht, Netherlands; ^2^Department of Clinical Psychology, Utrecht University, Utrecht, Netherlands; ^3^PSYTREC, Bilthoven, Netherlands; ^4^Academic Centre for Dentistry Amsterdam (ACTA), University of Amsterdam, VU University Amsterdam, Amsterdam, Netherlands; ^5^School of Health Sciences, University of Salford, Manchester, United Kingdom; ^6^Institute of Health and Society, University of Worcester, Worcester, United Kingdom

**Keywords:** PTSD, EMDR, emotional memories, trauma, Flash technique

## Abstract

**Introduction:** The Flash technique is a novel intervention aimed at rapidly decreasing the subjective disturbance of an aversive memory, thereby serving as a potential way of treating post-traumatic stress disorder (PTSD). The protocol is used to stimulate clients to engage in positive imagery while being discouraged to actively recollect the targeted disturbing memory. Previous research into the Flash technique’s efficacy shows promising results, yet controlled studies are lacking.

**Objectives:** To test the efficacy of the Flash technique, it was compared to an abbreviated eye movement desensitization and reprocessing (EMDR) therapy protocol in a controlled experimental setting. We hypothesized that the Flash technique would lead to a larger decrease in the emotionality and vividness of an aversive autobiographical memory when compared to EMDR therapy. Our second hypothesis was that the procedure of the Flash technique would be evaluated more pleasant by its receiver.

**Method:** The sample consisted of 60 non-clinical participants (mean age = 25.28 years; 73.33% female) who were able to recall an aversive autobiographical memory. They were randomized to either the Flash technique or the EMDR therapy condition. Measurements consisted of emotionality and vividness-ratings pre and post intervention, and at 1-week follow-up.

**Results:** Bayesian analyses showed no differences between Flash and EMDR to the extent to which the emotionality and vividness of their memory was reduced. Afterward, the Flash technique was rated more pleasant than EMDR.

**Conclusion:** The results support the claim that the Flash technique might be used as a brief and efficacious intervention for individuals suffering from disturbing memories. Although the results suggest that its efficacy does not differ from EMDR, the Flash technique seems to yield similar outcomes in a more pleasant way. Further research into its working mechanisms and in a clinical sample is required.

## Introduction

The treatment of post-traumatic stress disorder (PTSD) is a well-studied topic, having led to the development of global treatment guidelines aiming to maximize the therapeutic effectiveness of individuals suffering from this often debilitating mental health condition (American Psychiatric Association, 2017; International Society for Traumatic Stress Studies, 2018; National Institute for Clinical Excellence, 2018). However, many individuals suffering from PTSD still do not respond to the evidence based treatments from the guidelines or experience only moderate effects, whereas dropout and relapse often occur (Schottenbauer et al., 2008; Imel et al., 2013). Furthermore, treatment costs remain high (Mavranezouli et al., 2020). This situation prompts clinicians and researchers to explore ways to cost-effectively improve the effectiveness of treatments for PTSD. This urge to enhance the (cost-)effectiveness of these treatments brings forth a variety of new techniques and therapeutic interventions. Although the introduction of new therapies stimulates the constant development of a field, these interventions should remain subject of scientific examination before being employed in clinical practice.

One novel trauma-related intervention is called the Flash technique, a brief therapeutic procedure aimed to rapidly alleviate the distress of a disturbing memory (Manfield et al., 2017). A core feature of this technique is the engagement in positive imagery (e.g., an enjoyable activity, a wonderful memory, a dear person, or someone’s favorite music), while patients are discouraged from intentionally activating the targeted traumatic memory. More specifically, upon target selection, patients are instructed to only briefly “touch” the memory and rate its disturbance. Subsequently, patients are regularly asked to just lightly check in on the memory and notice any changes without tuning into the memory fully. The positive imagery is combined with recurrent blinking prompted by the therapist. Altogether, the intervention often takes less than 15 min to carry out. The Flash technique was originally developed as a type of “titration technique” preceding eye movement desensitization and reprocessing (EMDR) therapy. It was aimed at increasing the tolerability of recollecting a severely disturbing memory before treating it with EMDR therapy (Manfield et al., 2017). Currently, the Flash technique has evolved to a stand-alone trauma therapy. A detailed description is provided by Manfield et al. (2021).

Regarding the clinical effectiveness and safety of the Flash technique, a few scientific, peer-reviewed studies have been published. One consists of four case-studies in which the technique is being used as a preparation to EMDR therapy (Manfield et al., 2017). In this study, The Flash technique was applied by four different therapists with their own patients who met the diagnostic criteria of PTSD, although it was not specified whether an official clinical interview had been conducted. The application of the technique resulted in a reduction of both disturbance of the targeted aversive memory and avoidance, possibly allowing for subsequent successful EMDR therapy. In one case, it appeared that additional EMDR therapy was no longer needed. Another series of case studies describes the Flash technique applied as group therapy for the treatment of multiple disturbing memories among five addicts in a homeless shelter who suffered from PTSD-related and dissociative symptoms upon recollection of the memory (Wong, 2019). The therapy consisted of eight 50-min group sessions preceded by an individual training session. Although PTSD criteria were not assessed with an official clinical interview, the results showed a large decrease in self-reported PTSD symptoms, subjective distress, and symptoms of depression and dissociation. A more recent uncontrolled study investigated the effects of the Flash technique being applied online. The sample consisted of a large group of healthcare workers (*N* = 175) who participated in a 1-h webinar to reduce disturbance experienced from a memory of working with COVID-19 patients (Manfield et al., 2021). The webinar consisted of 30 min of psychoeducation, followed by two 15-min group sessions, during which either one memory was treated twice or two different memories were targeted. Emotionality of the memory, as indexed by subjective units of disturbance (SUD), showed a large decrease after undergoing the intervention.

Despite the promising results of previous, uncontrolled studies using the Flash technique, this intervention has not been tested using a controlled design with random allocation. Therefore, the claim made by the authors that “The flash technique (FT) is a low-intensity individual or group intervention that appears to rapidly lessen the distress of disturbing and traumatic memories” (Manfield et al., 2021, p. 1) should be interpreted with caution. This was the reason that we conducted an experimental, lab-analog RCT to provide valid data which may serve as a good starting point for any potential further research into this therapeutic intervention. More specifically, we sought to determine the efficacy of the Flash technique in decreasing the disturbance related to aversive memories. Because the Flash technique is often presented in relation to EMDR therapy, an evidence-based therapy for PTSD (De Jongh et al., 2019; Mavranezouli et al., 2020), and because the Flash technique shows some procedural similarities (in terms of memory recollection alternated with cognitive demanding tasks), we compared the effects of the Flash technique to EMDR therapy using a sample from a non-clinical population. Based upon the previously described case studies, we hypothesized that the Flash technique would be more effective in reducing the emotionality and vividness of an autobiographical disturbing memory compared to EMDR therapy when used as a brief intervention. Another promising aspect of the Flash technique might be its tolerability as the technique capitalizes on maintaining a positive focus. Accordingly, we hypothesized that the Flash technique would be rated more pleasant upon completion by the participant when compared to EMDR therapy.

## Materials and Methods

### Participants

We recruited participants of 18 years and older with sufficient command of the Dutch language, access to a quiet room, in possession of a computer or laptop with a stable internet connection for using a web-based application for the therapy, and who were able to recall a disturbing memory. They were recruited through social media posts and subsequently screened for participation. Exclusion criteria were the following: a SUD score lower than 6 upon recollection of the disturbing memory, a current diagnosis of a depressive disorder, bipolar disorder, PTSD, psychosis, or autism spectrum disorder, current use of antidepressants, mood stabilizers, benzodiazepines or antipsychotics, current treatment for psychiatric symptoms, prior EMDR therapy for 10 or more sessions and/or less than 3 years ago, visual or auditory impairments, and alcohol or drug use less than 12 h before study participation.

To our invitation responded seventy-nine people of whom 19 were excluded from participation or data analyses for a variety of reasons: seven received EMDR therapy less than 3 years before participation, four were not able to establish a stable video call connection with the researcher, three canceled their appointment after initial application, two gave a SUD rating lower than six upon recollection of their disturbing memory, one showed PTSD symptoms during participation, one used alcohol less than 12 h before participation, and one’s data were not stored due to technical issues. Therefore, data were analyzed from a total of 60 participants. They had a mean age of 25.28 years (SD = 4.67), and 73.33% were female. Participants were reimbursed with either course credits or financial compensation independent of study completion.

Although Bayesian statistics do not require an exact a piori power analysis, one was preregistered on OSF^1^ to guide recruitment numbers. The analysis suggests a total sample size of *N* = 86. Due to the exclusions and depletion of study resources, this number was not met. Yet, the current sample size of an average of 30 participants per group is deemed sufficient to detect expected statistical differences.

### Materials

#### Emotionality

Participants rated the subjective disturbance upon recollection of the aversive memory using the SUD-scale (Subjective Units of Disturbance; Wolpe, 1969). The scale ranges from 0 (no distress at all) to 10 (maximum distress). It has good psychometric qualities and is considered the standard outcome measurement in EMDR research, as well as EMDR therapy in clinical practice (Kim et al., 2008; Shapiro, 2018). In the current study, SUD-scores were assessed verbally by the researchers pre- and post intervention and at 1-week follow-up.

#### Vividness

The vividness of the disturbing memory was rated on a 11-point Likert scale ranging from 0 (not vivid at all) to 10 (extremely vivid). This measure is commonly used in experimental EMDR related research (e.g., Van den Hout and Engelhard, 2012). Vividness ratings were assessed orally by the researchers pre- and post intervention and at 1-week follow-up.

#### Treatment Evaluation

The researchers asked the participants to evaluate the procedure after completion of the experiment: “How pleasant did you find this procedure, estimated on a scale ranging from 0, “not pleasant at all,” to 10, “very pleasant”?”

#### Pleasantness of the Positive Memory

The pleasantness of the positive memory in the Flash condition was measured during the intervention by verbally asking the participant: “How pleasant do you score this memory now on a scale ranging from 0 to 10?”

### Procedure

Study procedures were ethically reviewed and approved by the Faculty Ethics Review Board of the Faculty of Social and Behavioral Sciences, Utrecht University (UU; Registration ID: 20-0227). Two graduate students of the Clinical Psychology Master’s program of UU conducted the experiment. All study procedures were performed online because face-to-face testing was not possible in the faculty labs due to restrictions regarding the COVID-19 pandemic. Participants applied by sending an email to the researchers, after which they were called to explain study procedures, screened for in- and exclusion criteria and when seemingly eligible, an online appointment was scheduled. Then, they were sent an email containing appointment details, instructions for video calling using the web-based EMDR application “EMDR Platform” (including the possibility for regular one-on-one video conferencing; EMDR Platform, 2020), the information letter, and a link to the online informed consent form. The letter contained detailed study information regarding the procedure, voluntary participation, possible risks and disadvantages, reimbursement, anonymity and confidentiality of the data, and contact information of the researchers.

At the commencement of the online appointment, the researcher checked the quality of the video calling connection and made sure participants sat in a quiet room where they would not be disturbed. They asked whether the participant read the information letter and answered any remaining questions. Participants were then instructed to sign the online informed consent form using the previously sent email link. Next, they were screened for exclusion criteria. When included, the pre SUD and vividness measurement of the aversive memory was conducted, followed by the randomization into either of the Flash or EMDR protocolized treatment procedures and subsequent post measurement. Participants were allocated randomly in a condition by order of inclusion, using a randomly generated list of participant IDs connected to a condition. The number of tested conditions was counterbalanced between the researchers. All screening and measurement data were collected orally by the researchers and instantly registered in the online, university supported, survey tool Qualtrics (2020). Since the researchers conducted both the intervention and the measurements, they were not blinded to the condition. After completing the post measurement, perceived pleasantness of the treatment was measured and some additional open-ended questions regarding their experience of the procedure were asked. Next, the researcher scheduled a telephonic follow-up appointment and concluded the online appointment. The follow-up appointment consisted of the verbal follow-up measurement of SUD and vividness. After completion, participants were debriefed, reimbursed and thanked for their participation.

### Treatment

The interventions were conducted online by two graduate students in clinical psychology, trained and supervised in the procedures by two of the authors (AJ and SM), who were trained in the procedure by attending a workshop and an online training by the originator of the Flash technique. Eight-min protocols were used, either an abbreviated version of the EMDR standard protocol (Ten Broeke et al., 2019), or the Flash technique protocol (De Jongh and Matthijssen, 2020; Manfield et al., 2021). Protocol adherence was ensured by evaluating video recordings of trial sessions. Both interventions were preceded and followed by SUD and vividness measurements of the aversive memory.

#### Eye Movement Desensitization and Reprocessing

The procedures of EMDR therapy are standardized in an eight-phase protocol (Shapiro, 2018; De Jongh and Ten Broeke, 2019; for a description).^2^ In the current study, EMDR therapy was conducted using the web-based EMDR application “EMDR Platform” (EMDR Platform, 2020). The application allows its user to conduct eye movements by controlling the speed of a horizontally moving dot. Meanwhile, the therapist is able to see the participant allowing adherence to the task to be checked. The participant is not able to see the therapist, seeing only the dot on a neutral full-screen background of the application. EMDR therapy started with a practice set of eye movements, adapting the movement frequency to personal maximum speed. Subsequently, most of the assessment phase of the EMDR standard protocol was applied, including selecting and rating the most disturbing image of the aversive memory and focusing on emotions and physical sensations. Next, while stimulated to keep the most disturbing image in mind, the participants performed a set (30 s) of eye movements after which they were asked to report upcoming associations. These sets were repeated until they reported similar or no associations two subsequent times. Consequently, the therapist went back to the most disturbing image to evaluate treatment progress by assessing the SUD, before continuing with a new series of sets. This process was repeated until a SUD score of zero was achieved, or until the maximum session time of 8 min was over.

#### The Flash Technique

The procedures of the Flash technique are described in the protocol by Manfield et al. (2021). In the present online study, the Flash technique was provided using the video calling function of a web-based EMDR application “EMDR Platform” (EMDR Platform, 2020). The intervention started with the target selection for the positive imagery. Hereby, the participant was instructed to recall the positive memory of an activity, person, animal, vacation, music, or whatever induced an immediate positive emotion and/or laughter. Then, one set of “Flash” was practiced, wherein the researcher prompts the participant by saying the word “Flash” to perform three emphatic and quick blinks, while the participant was also instructed to not think about the disturbing memory. Subsequently, the positive memory was recalled by stimulating vivid recollection, activating sensory details, and rating its pleasantness. Participants were then asked to engage in the positive imagery, while the therapist cued them repeatedly to perform Flash sets for five times. Consequently, the therapist evaluated treatment progress by asking whether or not any change occurred in the memory and to rate its SUD before starting a new round of positive imagery and Flashes. This process was repeated until a SUD score of zero was achieved, or until the maximum intervention time of 8 min was over.

### Design

The study used a two (Condition: Flash, EMDR) by three (Time: pre, post, follow-up 1) mixed design. The independent variable Condition was measured between-subjects and was either the Flash Technique or EMDR treatment. The within-subjects variable Time comprised of SUD and vividness measurements prior to treatment (pre), directly following completion (post), and at 1-week follow-up (follow-up 1). Additionally, pleasantness of the procedure was rated as dependent variable.

### Data Analysis

Statistical procedures were preregistered on OSF (see text footnote 1). All data were analyzed by Bayesian methods with the statistical software JASP (v0.14.1; JASP Team, 2020). In Bayesian statistics, the Bayes Factor (BF) is computed and used to express the data’s relative support for one hypothesis or model vs. one or multiple others. A BF > 1 indicates support for the proposed hypothesis or model, with larger values representing more support. A BF < 1 indicates support for the null hypothesis or alternative model(s), with smaller values representing more support. BF values close to 1 indicate equal support. The advantage of Bayesian statistics compared to Null Hypothesis Significant Testing (NHST) is the absence of a strict cut-off value (e.g., *p* < 0.05) on which the evaluation of the true or falseness of a hypothesis is based. Notwithstanding, a general indication on how to interpret the BF is expedient: BFs of 1–3 are considered minor support, BFs of 3–10 indicate moderate support, and BFs > 10 represent major support.

Bayesian repeated measures analyses of variance (ANOVAs) were conducted to analyze overall group differences in treatment efficacy, with condition (Flash, EMDR) as a between-subjects variable and SUD and vividness ratings representing the within-subject variable time (pre, post, follow-up). *Post hoc* analyses of slope differences consisted of Bayesian Independent Samples T-Tests (ISTTs) with condition as the independent variable and SUD and vividness difference scores (pre-post, pre-follow-up, post-follow-up) as dependent variables. For other single measurement analyses such as randomization checks or treatment evaluation, ISTTs were used.

Analyses of variance outcomes are reported using the notation BF_m_, which quantifies the support the data shows for one model compared to all other tested models. In this study specifically, these models consist of the main effects for Condition and Time, the interaction effect, as well as the combination between these effects. BF_m_ is computed by dividing the posterior odds of the tested model by the average posterior odds of the other models. ISTT outcomes are reported using the notation BF_10_, thereby expressing the relative support of the tested hypothesis vs. the null hypothesis. When the null hypothesis is supported instead, the notation BF_01_ is used. Default priors were used for all analyses (Rouder et al., 2012). JASP automatically corrects for multiple testing by fixing to 0.5 the prior probability that the null hypothesis holds across all comparisons (Westfall et al., 1997).

## Results

### Descriptive Statistics

Data from 60 participants were included in the analyses. One participant could not be reached for the follow-up measurement, leading to one missing value for both SUD and vividness at this time point. Participants rated their disturbing memory with an average SUD of 7.63 (SD = 0.80) at baseline. The average baseline vividness score of the memory was 8.03 (SD = 1.43). In two cases, a SUD score of 0 was reached before the 8-min session time was over, although the remaining time in both cases was less than 30 s. In the Flash condition the pleasantness of the positive memory was rated with an average of 8.94 (SD = 0.83) at baseline, which did not differ from follow-up (*M* = 9.44; SD = 2.08) as shown by a Bayesian ISTT (BF_01_ = 2.06).

### Randomization Check

There were no differences between the two conditions at baseline for SUD, as shown by Bayesian ISTT (BF_01_ = 3.79). Considering vividness, the model including differences between the conditions was supported marginally (BF_10_ = 1.46), suggesting a higher baseline vividness score in the Flash condition (*M* = 8.38, SD = 1.39) when compared to the EMDR condition (*M* = 7.64, SD = 1.39). Successful randomization of age (Bayesian ISTT; BF_01_ = 1.90) and gender (Bayesian contingency table; BF_01_ = 2.04) was supported. Differences in therapeutic effectiveness were analyzed by comparing the differences in SUD and vividness score reductions from pre to post. A Bayesian ISTT showed large support for a difference in SUD decrease between both researchers (BF_10_ = 522.91). Vividness did not reduce differently (BF_01_ = 3.00). However, both experimenters tested an equal number of subjects in both conditions (Bayesian contingency table; BF_01_ = 2.43). Therefore, corrections in the analyses were deemed redundant.

### Efficacy

#### Emotionality

**FIGURE 1:**
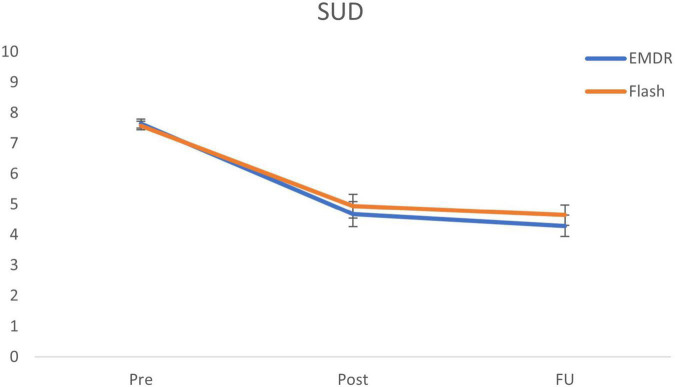
Mean (SE) subjective units of disturbance (SUD) scores for all time points specified per condition. EMDR = eye movement desensitization and reprocessing; SUD = subjective unit of disturbance; and FU = follow-up after 1 week.

The Bayesian repeated measures ANOVA comparing emotionality between conditions (Flash, EMDR) and over time (pre, post, follow-up) shows the most support for the model including only a main effect of Time (BF_m_ = 11.78). This main effect is further specified by *post hoc* tests showing major support for a decrease in SUD ratings from pre to post (BF_10_ = 9.75 × 10^11^; Cohen’s d = 1.35), and pre to follow-up (BF_10_ = 4.13 × 10^16^; Cohen’s d = 1.77). No support was found for differences between post and follow-up (BF_01_ = 2.30). The alternative ANOVA model including the main effect for Time and Condition receives no convincing support (BF_m_ = 1.15). Furthermore, the analysis shows strong evidence against the model including the interaction effect between Time and Condition (BF_m_ = 0.13). This outcome is further supported by the *post hoc* ISTTs comparing the differences in SUD decreases between conditions, showing support for the null models (pre-post: BF_01_ = 3.63; pre-follow-up: BF_01_ = 2.67). For an overview of the SUD ratings for all time-points, see Figure 1.

#### Vividness

**FIGURE 2:**
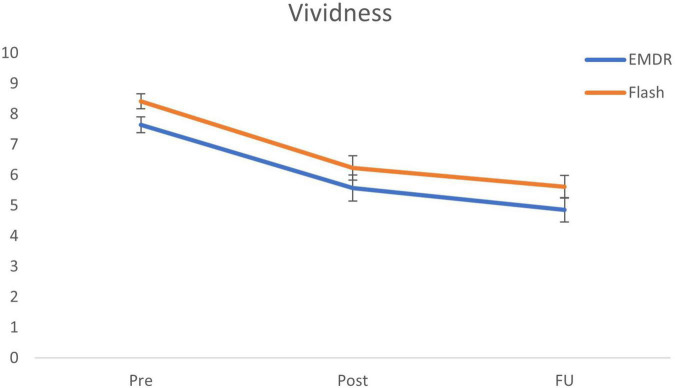
Mean (SE) vividness scores for all time points specified per condition. EMDR = eye movement desensitization and reprocessing; and FU = follow-up after 1 week.

The Bayesian repeated measures ANOVA with Condition (Flash, EMDR) as between-subjects variable and vividness ratings representing the within-subjects variable Time (pre, post, follow-up) shows roughly equal support for the model including a main effect of Time and Condition (BF_m_ = 3.82), and the model including only a main effect of Time (BF_m_ = 3.41). *Post hoc* tests further specify this main effect by providing major support for a decrease in vividness ratings from pre to post (BF_10_ = 8.32 × 10^6^; Cohen’s d = 0.94), pre to follow-up (BF_10_ = 1.91 × 10^13^; Cohen’s d = 1.46), and moderate support for a decrease from post to follow-up (BF_10_ = 4.81; Cohen’s d = 0.36). The ANOVA shows strong support against the alternative model including the interaction effect between Time and Condition (BF_m_ = 0.22). *Post hoc* ISTTs further support this outcome by showing support for the null model including no differences in vividness decreases between conditions (pre-post: BF_01_ = 3.77; pre-follow-up: BF_01_ = 3.78). For an overview of the vividness ratings for all time-points, see Figure 2.

### Treatment Evaluation

Differences in treatment evaluation between conditions were analyzed using a Bayesian ISTT. The analysis showed moderate support for the model with a difference between conditions (BF_10_ = 7.99). More specifically, participants gave their treatment experience a more pleasant rating in the Flash condition (*M* = 8.25; SD = 2.60) when compared to the EMDR condition (*M* = 6.61, SD = 1.57; BF_10_ = 7.99).

## Discussion

The current study had two objectives. The first was to determine the efficacy of the Flash technique in reducing the emotionality and vividness of a disturbing autobiographical memory when compared to an abbreviated EMDR therapy protocol in a non-clinical sample. The hypothesis that the Flash technique would me more efficacious as a brief intervention was not supported by the results. More specifically, we could not detect any differences between the Flash and EMDR condition regarding decreases in either emotionality or vividness from pre- to post-intervention as well as at 1-week follow-up. The second objective was to compare the pleasantness of the procedure between both conditions. The analyses revealed that participants evaluated the Flash technique to be more positive post-treatment, thereby providing support for the second hypothesis.

The results regarding the efficacy of the Flash technique are in accordance with previous studies showing that the Flash technique has a large effect on decreasing the subjective disturbance of an aversive memory (Manfield et al., 2017, 2021; Wong, 2019). Although Manfield et al. (2017) introduced the Flash technique as a preparation to EMDR therapy, the current study shows it might even be efficaciously used as a standalone intervention. It is important to note that most of the earlier support for the claim that the Flash technique shows positive effects on aversive memories is based upon research using a group intervention protocol (Wong, 2019; Manfield et al., 2021). The Flash technique might have the particular advantage over other more trauma-focused interventions in that the participants do not have to share the content of their trauma memories with others, not even with the therapist (”blind to therapist”); in fact, just following process instructions seems sufficient in this context. Although a group setting might be favorable in terms of cost-effectiveness, in clinical practice the treatment of PTSD will still be applied largely through individual sessions. Nevertheless, future randomized controlled research should further evaluate the Flash technique’s applicability and effectiveness as a group therapy.

Regarding our second study aim, the finding that the Flash technique was evaluated more positive post-treatment than EMDR therapy is in line with suggestions made in previous studies, albeit the pleasantness or tolerability of the Flash technique were not explicitly tested in these studies. Given that the Flash technique seems easily applicable to individuals with complex dissociative symptoms (Manfield et al., 2017; Wong, 2019), the current results are of importance. By definition and in essence, trauma-focused therapy is an unpleasant procedure since it involves the active recollection of fearful memories that were previously avoided, and therefore, a less intrusive and even pleasant form of treatment might have a positive effect on dropout rates (Imel et al., 2013). Moreover, a more tolerable treatment for the client could also connote less burden on the therapist, as well as a decrease of other negative side-effects of trauma-focused treatments such as secondary therapist traumatization (Canfield, 2005).

How can the finding that we could not detect any difference between both treatments in reduction of both emotionality and vividness of participants’ disturbing memories be explained? Answers might be found when examining potential and supposed working mechanisms of both therapies. First of all, the engagement in positive imagery as applied during the Flash technique could be considered a form of counterconditioning. This mechanism was shown to be effective in decreasing fearful stimuli in lab experiments (e.g., Kang et al., 2018), is proven to be an effective technique in the treatment of anxiety and trauma-related disorders (e.g., Newall et al., 2017; Daneshvar et al., 2021), and is part of several successful treatment protocols for these mental health conditions such as Competitive Memory Training (COMET) and Visual Schema Displacement Therapy (VSDT; Staring et al., 2016; Matthijssen et al., 2019, 2021b). Secondly, one could explain the effects of both EMDR therapy and the Flash technique based upon the working memory theory. To this end, there is mounting quantitative support for this account which predicts that due to its limited capacity, for humans’ working memory it is difficult to hold a disturbing memory in mind while simultaneously performing a dual task (e.g., actively conducting rapid eye movements), leading to a reduction in emotionality and vividness of the disturbing memory (Gunter and Bodner, 2008; Van den Hout and Engelhard, 2012; De Jongh et al., 2013). Subsequently, the memory reconsolidates in this altered way (Schwabe et al., 2014). In accordance with the working memory theory, the positive imagery and blinking as part of the Flash technique might also be just another way of dual tasking. From this perspective, boosting the competition between the tasks should increase the effectiveness of the intervention, as is one of the fundamental explanations for proposed improvements to EMDR therapy (i.e., “EMDR 2.0”; Matthijssen et al., 2021a). However, this suggests that activation of the memory is pivotal and should therefore be maximized, speaking against the discouragement of memory activation in the Flash technique. Contrary to the evidence supporting maximum memory activation, neuroscientific research shows that reconsolidation not only occurs in the period directly following recollection of a memory, but appears to continue in the following weeks (Kida, 2019). This suggests that changes in memory not only occur during conscious activation, but may continue unconsciously, and that a brief activation of a memory, as applied in the Flash technique, might be sufficient to subsequently effectuate alteration of its subjective disturbance without further activation. The absence of activation combined with positive imagery in the Flash technique might also be interpreted as a form of fear extinction. Contemporary models of classical conditioning theory predict that extinction occurs when a conditioned stimulus (CS) is presented in the absence of unpleasant consequences; that is, without occurrence of the unconditioned stimulus (US; Craske et al., 2014). During the Flash, after being confronted with an intense positive experience the patient is quickly turned to an aversive, seemingly threatening memory. The patient is still in a positive state and recalling the memory in his or her mind (CS) does not evoke an immediate aversive response (US), so that fear reduction can take place. In line with this, but viewed from another angle, neuroscientific research into subliminal exposure suggests that activation of the amygdala inhibits rapid reprocessing of a memory (Siegel and Weinberger, 2012; Siegel et al., 2020). These findings would advocate maximum deactivation of the amygdala during the treatment of aversive memories. Dual tasking might therefore be seen as just another way of deactivating the amygdala, a notion supported by a recent fMRI study (De Voogd et al., 2018). Taken together, the role of activation and subsequent dual tasking in order to achieve altered memory reconsolidation is an important area of future research.

Several limitations regarding the current study are worth mentioning. Firstly, the sample consisted of non-clinical participants recruited by student researchers. Although a non-clinical sample is commonly employed in a lab-based study into working mechanisms, future research should include a clinical, more heterogeneous, and larger sample to improve generalizability of the findings. Secondly, it was argued that a more positive treatment experience (as was shown in the Flash condition of the current study) might lead to fewer dropouts and thereby increase therapeutic effectiveness. However, the current lab-analog study contained a single 8-min session, rendering us unable to support this argument. Finally, the fixed treatment duration of 8 min in both conditions might have been too short to unveil differences in treatment effectiveness and efficiency. In only two of the 60 participants, a SUD score of zero was reached before treatment time was over, meaning that clinically speaking the treatment was not completed for the other participants. A study including longer sessions might differentiate in the number of SUD scores that reach zero, thereby differentiating in effectiveness (i.e., mean SUD decrease) and efficiency (i.e., mean required session time to reach a SUD score of zero). The major strength of our study is that it is the first randomized controlled trial using a procedurally standardized intervention protocol into the efficaciousness of the Flash technique. This lays a methodologically sound foundation for future clinical trials and studies into unraveling the Flash technique’s working mechanisms.

To conclude, the Flash technique was shown not to differ in efficacy from EMDR in a non-clinical sample, while being evaluated more positive by its recipients. Future research should focus on testing the Flash technique as a standalone treatment compared to a full-length, evidence based trauma-focused therapy in a patient sample diagnosed with PTSD. Such a study might substantiate claims made about the Flash technique being a more rapid and effective form of trauma-related treatment when compared to, for example, EMDR therapy. Furthermore, the absence of repeated memory activation and the use of positive imagery as part of the Flash technique suggest it might be valuable to further study the role of memory activation and positive imagery in the treatment of disturbing memories to the benefit of all PTSD treatments. Taken together, the introduction of the Flash technique might very well be an important next step on a path to more tolerable and thereby effective PTSD treatments.

## Data Availability Statement

The datasets presented in this article are not readily available because of privacy and ethical restrictions. Reasonable requests to access the datasets should be directed to the corresponding author.

## Ethics Statement

The studies involving human participants were conducted according to the guidelines of the Declaration of Helsinki, and reviewed and approved by the Ethics Committee of the Faculty of Social and Behavioral Sciences, Utrecht University (Registration ID: 20-0227; approval date: April 20, 2020). The participants provided their written informed consent to participate in this study.

## Author Contributions

TB, AJ, and SM: conceptualization and methodology. TB: software, investigation, data curation, writing – original draft preparation, visualization, and project administration. SM: resources and funding acquisition. AJ and SM: writing – review and editing. TB and SM: supervision. All authors have read and agreed to the published version of the manuscript.

## Conflict of Interest

AJ receives income from published books on EMDR therapy and for training postdoctoral professionals in this method. AJ and SM received income from a webinar on the Flash technique. The remaining author declares that the research was conducted in the absence of any commercial or financial relationships that could be construed as a potential conflict of interest.

## Publisher’s Note

All claims expressed in this article are solely those of the authors and do not necessarily represent those of their affiliated organizations, or those of the publisher, the editors and the reviewers. Any product that may be evaluated in this article, or claim that may be made by its manufacturer, is not guaranteed or endorsed by the publisher.

## Footnotes

1
https://osf.io/37zb9

2
https://www.emdria.org/about-emdr-therapy/experiencing-emdr-therapy/
